# Effect of kinesio taping intervention on the muscle strength and balance of college basketball players with functional ankle instability

**DOI:** 10.3389/fphys.2023.1064625

**Published:** 2023-03-29

**Authors:** Rui Li, Rui Qin, Yajun Tan, Hengxian Liu, Kun Wang, Liang Cheng

**Affiliations:** ^1^ School of Sports Medicine and Health, Chengdu Sport University, Chengdu, China; ^2^ Sport Hospital, Chengdu Sport University, Chengdu, China; ^3^ Shanghai University of Sport, Shanghai, China

**Keywords:** functional ankle instability, college basketball player, kinesio taping, isokinetic muscle strength, balance

## Abstract

**Objective:** The aim of this study was to investigate the effects of acute Kinesio Taping (KT) intervention on the muscle strength and balance ability of college basketball players with functional ankle instability (FAI).

**Methods:** Thirty college basketball players with FAI were treated with acute KT to test the changes in their muscle strength and balance ability.

**Results:** After acute KT intervention, the ankle dorsiflexion moment and the ankle plantar flexion moment increased by 34% and 19.9%, respectively. The stable plane test with the subjects’ eyes open decreased by 1%, whereas that with the subjects’ eyes closed decreased by 1.1%. The swaying environment test with the subjects’ eyes open increased by 2.4%. The swaying plane test with the subjects’ eyes open increased by 5.1%, whereas that with the subjects’ eyes closed increased by 16.2%. The swaying environment test with the subjects’ eyes open plus the use of a plane increased by 12.1%.

**Conclusion:** KT can increase the isokinetic strength of the ankle dorsum muscle and plantar flexion of college basketball players with FAI. The effect of KT in the static balance test was weaker than that in the dynamic balance test. The findings indicate that KT can significantly improve the balance ability of college basketball players with FAI during dynamic sports.

## 1 Introduction

An ankle sprain is the main ankle injury in sports. The incidence of ankle injury is 76.7%, accounting for 10%–17% of all joint injuries in college athletes (Kim et al., 2022; Serra-Añó et al., 2021; D'Hooghe et al., 2020). Lateral ankle joint injury is one of the most common injuries (Doherty et al., 2017; Herzog et al., 2019; Alshahrani and Reddy, 2022). Studies have shown that functional ankle instability (FAI) is common in people who play jumping and turning sports, such as basketball, volleyball or soccer (Lin et al., 2021a). Approximately 70%–80% of basketball players develop FAI after their first ankle sprain (Santos and Liu, 2008). FAI leads to decreased postural control ability, which manifests as poor muscle strength (Mao et al., 2021), balance (Doherty et al., 2014), proprioception (Alghadir et al., 2020; Xu et al., 2022) and neuromuscular control (Huang et al., 2021). Falling may also increase the risk of a second sprain (Simpson et al., 2019).

Kinesio Taping (KT) was invented by Dr. Kenso Kase in Japan (Williams et al., 2012). The application of KT supports and relaxes muscles, ligaments and fascia, as well as other soft tissues. Tai Chi combined with KT significantly improves the dynamic and static balance ability of FAI football players (Li et al., 2022a). Furthermore, KT increases dynamic postural control in FAI patients after ankle muscle fatigue (Li et al., 2022b). KT application also improves the lower limb stability of patients with chronic ankle instability (CAI) (Yu et al., 2021). However, some studies suggest that KT has no positive effect on postural control and functional activities (Nunes et al., 2021).

Injuries to the ankles are usually caused by inversion (Woods et al., 2002; Doherty et al., 2014). Hence, increasing the strength around the ankle joint can improve the dynamic postural stability of the body during landing movement (Dewar et al., 2019). Increasing ankle muscle strength *via* KT intervention may reduce the recurrence of sprain in basketball (Shilun, 2011). KT application to college basketball players with FAI has recently been the focus of muscle strength and balance ability research, and the existing research on KT on muscle strength and balance ability is still controversial. The results of some studies indicate that KT does not seem to increase muscle strength or improve the balance ability of subjects (Shields et al., 2013; Bailey and Firth, 2017; Martonick et al., 2020). However, other studies have shown that KT plays a certain role in improving the muscle strength and proprioception of injured people (Williams S et al., 2012; Lin et al., 2021b; Mao et al., 2021; Biz et al., 2022). In this study, the aforementioned research scope is clarified by analysing the changes in muscle strength and balance ability through KT intervention and then evaluating its effect on the ankle of college student basketball players with FAI. The KT intervention was also conducted to improve muscle strength and balance ability and consequently derive the application’s theoretical basis. The research hypothesis is stated as follows: KT intervention can improve the ankle isokinetic muscle strength and balance ability of college basketball players with FAI.

## 2 Materials and methods

### 2.1 Research object

This study was approved by the Human Testing Ethics Committee of Chengdu Sport University [2022] No. 3, and 30 college basketball players who met the standards were selected for this research. The age range of the subjects was 21.5 ± 1.2 years, their height range was 179.33 ± 7.56 cm, and their weight range was 78.9 ± 9.76 kg. Regarding the Cumberland CAI tool (CAIT), the left side was 24.07 ± 6.27, and the right side was. 22.4 ± 5.59), with 13 and 17 patients presenting left ankle points (≤24) and right ankle points (≤24), respectively.

### 2.2 Selection of samples

The diagnostic criteria were as follows (Santos and Liu, 2008): 1) two or more unilateral varus sprains of the ankle joint; 2) subjective ankle instability that occurred twice or more in the past 6 months; and 3) negative anterior drawer test of the talus.

Included in the standard were the following items (Delahunt et al., 2010; Rein et al., 2020): 1) repeated ankle sprain more than twice; 2) aged 18 to 25; 3) most recent sprain was 30 days ago; 4) completed the ankle instability questionnaire by using CAIT (Wright et al., 2017), with a score of ≤24 points for the unilateral ankle joint; 5) a negative result in the anterior ankle drawer test; 6) no history of fracture or surgery of the lower limbs; and 7) informed that the study was based on the Declaration of Helsinki, with informed consent forms signed.

The exclusion criteria were as follows (Kunugi et al., 2018): 1) evident dislocation and fracture; 2) simultaneous severe sprain of both ankle joints; 3) other neurological diseases that may affect balance and muscle strength; and 4) skin allergy to KT.

### 2.3 Research methods

#### 2.3.1 Experimental method

**FIGURE 1:**
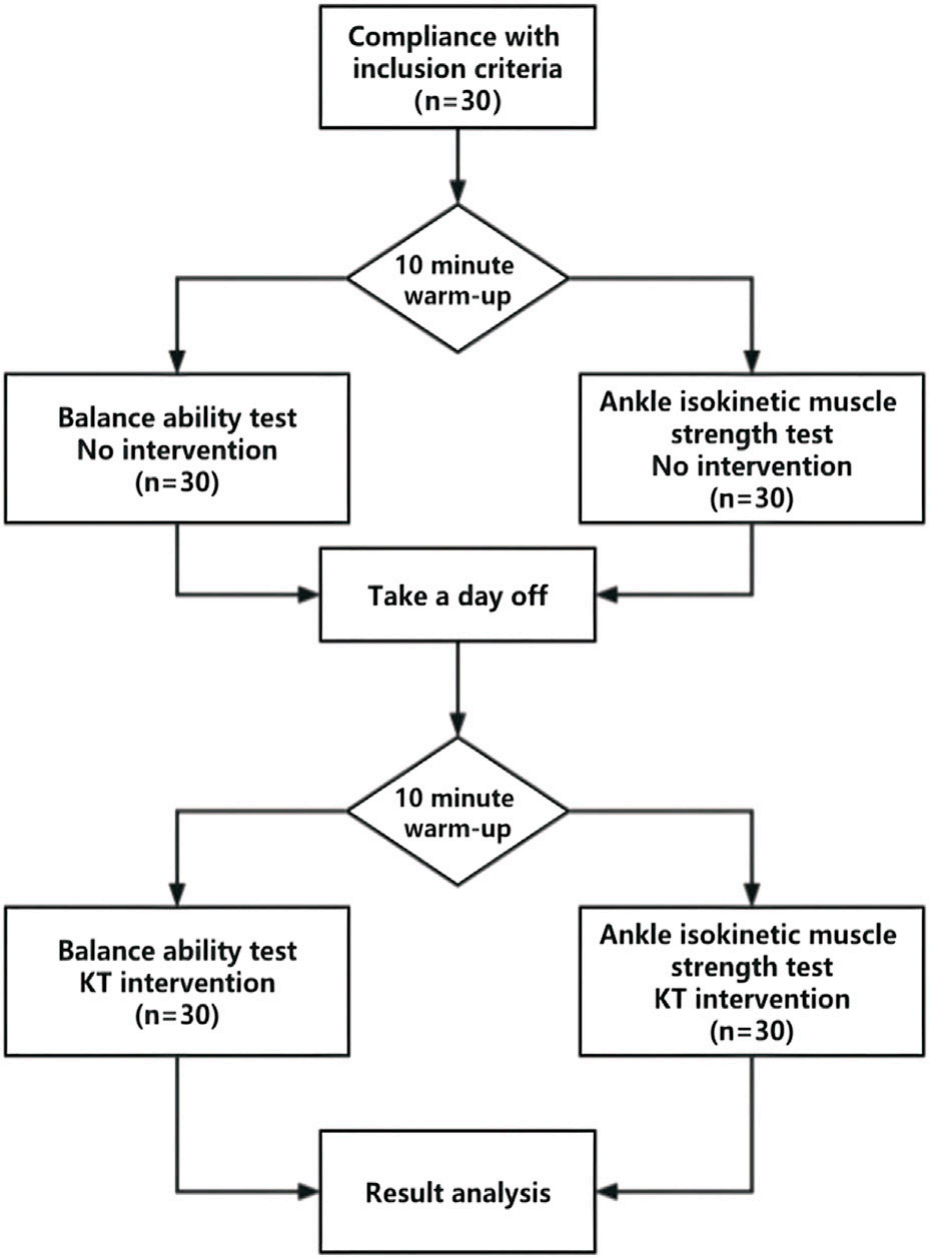
Experimental design flow chart.

Subjects were tested before and after the KT intervention. In the first experiment (i.e., before the KT intervention), the balance ability and lower limb muscle strength of all subjects were tested. In the second experiment (i.e., after KT intervention), the balance ability and lower limb muscle strength of all subjects were tested. The interval between the two experiments was 1 day, during which the subjects were required to maintain normal sleep and were not allowed to perform any muscle strength or balance training of the lower limbs (Figure 1).

#### 2.3.2 Stick firm method

**FIGURE 2:**
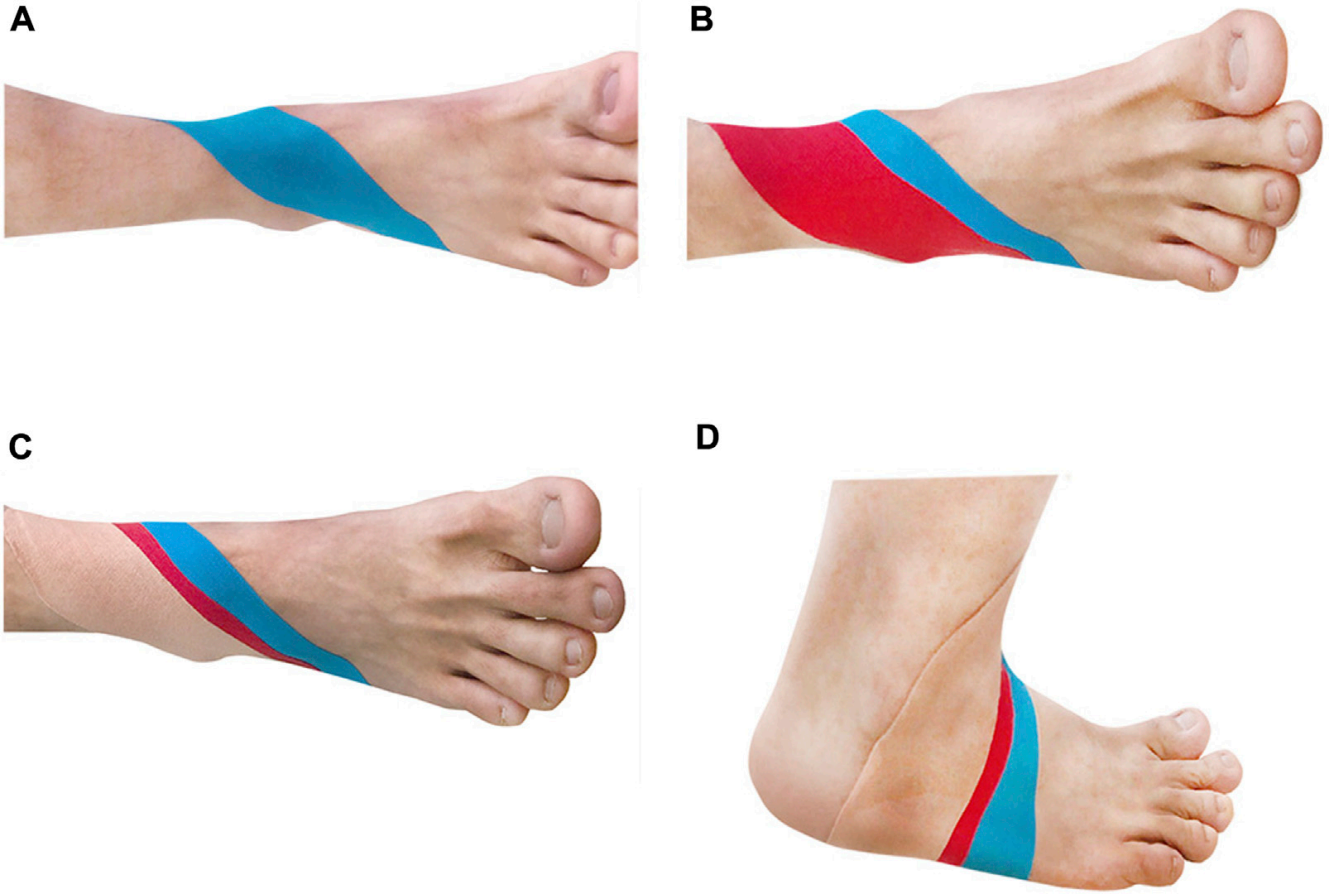
KT group **(A)** The first KT; **(B)** The second kT **(C)** The third KT; **(D)** Schematic diagram after completion.

According to John et al. (2018), for the first KT intervention, the anchor point should be set below the medial malleolus to prevent ankle sprain. In this method, an experimenter stretches the KT forward and pastes it along the position of the talus whilst simultaneously pressing the subject’s thigh against the ball of the foot (i.e., the subject’s foot is in a dorsiflexion position). Moderate stretching tension (approximately 30%) was applied to the KT through the lateral malleolus and terminated at the sole (Figure 2A). For the second kT intervention, the experimenter reversed pastes the KT along the first path with maximum tension (approximately 50%). The KT, passing from the outside of the foot, is stretched along the front of the ankle, and the pasting is terminated at the inside of the tibia (i.e., the KT should be one-third shorter than the original measurement length) (Figure 2B). If necessary, a third KT intervention may be adopted. The anchor point is set at the bottom of the foot. Without any tension to the KT, the experimenter, starting from the middle position, passing from the outside and front of the foot and bypassing the ankle joint, places the tail-end of the KT on the inside of the tibia. Then, the other end of the KT is placed by the experimenter on the inside of the foot, straining through the medial malleolus, then pulling back and terminating at the end of the first 2 KTs. The third KT intervention is usually conducted to allow the first and second kT interventions to fit well into the skin (Figure 2C). Figure 2D presents an image of a completed KT intervention.

#### 2.3.3 Balance ability test

The Smart Equi Test balance training tester developed in the United States was used, and the sensory integration experiment (SOT) was selected to perform six tests, which can be described as follows: 1) subjects with eyes open and floor and visual environment stable; 2) subjects with eyes closed and floor stable; 3) subjects with eyes open, floor stable and visual environment mobile; 4) subjects with eyes open, floor mobile and visual environment stable; 5) subjects with eyes closed and floor mobile; and 6) subjects with eyes open and floor and visual environment mobile. The tests were scored between 100 points (stable) and 0 points (fall). The balance ability index was taken as the test index; the higher the score is, the stronger the balance ability (Pickerill and Harter, 2011). Table 1 presents a detailed overview of the SOT (Delahunt et al., 2010).

**TABLE 1:** Overview of the six SOT tests.

Test project	Visual environment		System response	
	Eyes	Floor	Prejudice	Apply
1	Open	stable	—	proprioception
2	Closed	stable	visual sense	proprioception
3	Open	stable	visual sense	proprioception
4	Open	instability	proprioception	visual sense and vestibular
5	Closed	instability	Proprioception and visual sense	vestibular
6	Open	instability	Proprioception and visual sense	vestibular

Note: Test 1: Eyes Open, Floor and Visual Environment Stable; Test 2: Eyes Closed, Floor Stable; Test 3: Eyes Open, Floor Stable, Visual Environment Mobile; Test 4: Eyes Open, Floor Mobile, Visual Environment Stable; Test 5: Eyes Closed, Floor Mobile; Test 6: Eyes Open, Floor and Visual Environment Mobile.

#### 2.3.4 Ankle isokinetic muscle strength test

Flexion and extension tests (45°/s, 15 times) were performed on the affected ankle joints of 30 subjects by using the Swiss CONTRE isokinetic tester. The subjects were placed in the supine position during the test. Before the experiment, the subjects were allowed to warm up for 10 min. Then, the experimenter verbally stimulated the subjects to ensure that they could complete the whole experimental process as scheduled.

Peak torque was used as the test index. The maximum torque value of the joint muscle group, calculated during the ankle isokinetic test, was taken as the muscle strength level of the subject (Cheng et al., 2019; Cheng and Jiang, 2020).

### 2.4 Data statistics and analysis

The measurement data were processed as the mean ± standard deviation by using SPSS 20.0 statistical software. For measures conforming to normal distributions, the data of two measurements were tested by paired sample *t*-test, with the significance level set to *α* = 0.05.

## 3 Results

### 3.1 Isokinetic muscle strength of the ankle joint

After KT intervention, the peak moment of ankle dorsiflexion increased by 34% from 45.0 ± 22.3 to 60.3 ± 21.9 (*p* = 0.045). The moment of ankle plantar flexion increased by 19.9% from 48.8 ± 23.9 to 58.5 ± 22.3 (*p* = 0.039). The test results suggest the ability of the KT intervention to improve the ankle plantar flexion and dorsi flexion muscle strength of college basketball players with FAI. The difference was statistically significant (Table 2).

**TABLE 2:** Changes in isokinetic muscle strength of the affected ankle joint before and after KT intervention.

MG	NTPM (N·m)	KTPM (N·m)	PC (%)	t	*p*	95% CI
DF	45.0 ± 22.3	60.3 ± 21.9	34.0	−2.197	0.045^*^	−30.142 ∼ −0.364
PF	48.8 ± 23.9	58.5 ± 22.3	19.9	−2.223	0.039^*^	−18.814 ∼ −0.566

Note: MG, muscle group; NTPM, no taping intervention peak moment; KTPM, kinesio taping intervention peak moment; PC, percentage change; Nm, Newton m (moment unit); PF, plantarflexion; DF, dorsiflexion; Values are means ± standard deviation (SD); Significant differences (*p* < 0.05).

### 3.2 Balance of the ankle joint

According to the SOT test results, the equilibrium index of condition 1 decreased by 1% from 95.5 ± 1.2 to 94.5 ± 2.3 (*p* = 0.082), and the difference was statistically insignificant (Table 3). The balance index of condition 2 decreased by 1.1% from 93.3 ± 2.6 to 92.2 ± 3.0 (*p* = 0.208), and the difference was also statistically insignificant. The balance index of condition 3 increased by 2.4% from 92.3 ± 2.5 to 94.6 ± 2.3 (*p* = 0.002), with a statistically significant difference. These results indicate the ability of KT to improve the vestibular sensation of the ankle joint. In addition, the balance index of condition 4 increased from 85.6 ± 7.3 to 90.0 ± 4.9, which was 5.1% higher (*p* = 0.001), and the difference was statistically significant. Therefore, KT can improve the proprioception of the affected ankle joint. The balance index of condition 5 increased by 16.2% from 62.1 ± 17.9 to 72.2 ± 10.7 (*p* = 0.005), with a statistically significant difference. Hence, KT can significantly improve the proprioception of the affected ankle joint under visual deprivation conditions. The balance index of condition 6 increased by 12.1% from 73.1 ± 15.0 to 82.0 ± 5.5 (*p* = 0.006), and the difference was statistically significant. Thus, KT can improve balance ability even when both vestibular and proprioception are disturbed. In contrast, in conditions 1 and 2, the difference was statistically insignificant after intervention, as vision was not disturbed or only vision was shielded.

**TABLE 3:** Changes in balance ability before and after KT intervention.

Test project	PBI (score)	ABI (score)	PC (%)	*t*	*p*	95% CI
1	95.5 ± 1.2	94.5 ± 2.3	−1.0	1.871	0.082	−0.148–2.174
2	93.3 ± 2.6	92.2 ± 3.0	−1.1	1.321	0.208	−0.744–3.130
3	92.3 ± 2.5	94.6 ± 2.3	2.4	−3.883	0.002^*^	−3.684 ∼ −1.062
4	85.6 ± 7.3	90.0 ± 4.9	5.1	−5.405	0.001^*^	−6.211 ∼ −2.682
5	62.1 ± 17.9	72.2 ± 10.7	16.2	−3.309	0.005^*^	−16.70 ∼ −3.567
6	73.1 ± 15.0	82.0 ± 5.5	12.1	−3.242	0.006^*^	−14.76 ∼ −3.008

Note: PBI, Pre-intervention balance index; ABI: After-intervention balance index; PC, percentage change; Test 1: Eyes Open, Floor and Visual Environment Stable; Test 2: Eyes Closed, Floor Stable; Test 3: Eyes Open, Floor Stable, Visual Environment Mobile; Test 4: Eyes Open, Floor Mobile, Visual Environment Stable; Test 5: Eyes Closed, Floor Mobile; Test 6: Eyes Open, Floor and Visual Environment Mobile.

## 4 Discussion

The purpose of this study was to verify whether KT intervention can help improve the ankle isokinetic muscle strength and balance ability of college basketball players with FAI. After KT intervention, the performance of the subjects who participated in the ankle joint isokinetic muscle strength test significantly improved. In the static balance test, the difference in the results of the KT intervention was statistically insignificant. However, in the dynamic balance test, KT intervention significantly improved the balance ability of college basketball players with FAI.

### 4.1 Effect of KT on the ankle isokinetic muscle strength of college basketball players with FAI

This study verified the hypothesis that KT intervention can improve ankle isokinetic muscle strength in college basketball players with FAI. The obtained results showed a 34% and 19.9% increase in ankle extensor and flexor peak moments, respectively, which was similar to previous findings (Biz et al., 2022). Interestingly, although Muñoz-Barrenechea et al. (2019), reported that KT effectively improved the muscle strength of subjects with ankle instability, a few other researchers disagree. Nunes and de Noronha, 2019 applied KT to the calf triceps of 30 athletes and found no difference before and after the intervention. Ruoni (2021) found that KT could not significantly improve ankle dorsiflexion range of motion, lower limb muscle strength or subjective sensation in CAI patients within a short time. The conflicting results may be related to the different sticking methods and testing methods used by the researchers. In this study, the ability of KT to improve the isokinetic muscle strength of the subjects’ ankle joint may be explained by the elastic retraction force of the KT itself, producing traction on the subcutaneous tissue, thus causing neuromuscular stimulation and increasing muscle strength. On the basis of this analysed mechanism, KT may have enhanced the contraction ability of the damaged muscle, reduced the pain caused by muscle overextension, relieved the probability of spasm caused by muscle fatigue, improved the stability of the injured joint and surrounding soft tissue, prevented the abnormal work of the muscle from causing movement disorders of the normal joint and maintained the normal range of motion of the joint. These mechanisms can jointly reduce pain perception and increase muscle strength (Sarvestan and Svoboda, 2019). In addition, some researchers have reported the immediate effect of KT on muscle strength. For example, the tension of the pasting cloth can mechanically pull the muscle fascia, stimulate the contraction of weaker muscles and indirectly increase muscle strength (Merino-Marban et al., 2021; Li et al., 2022a).

### 4.2 Effects of KT on the balance ability of college basketball players with FAI

This study verified the research hypothesis that KT intervention can improve the balance ability of college basketball players with FAI. The equilibrium indices of conditions 3, 4, 5, and 6 increased by 2.4%, 5.1%, 16.2% and 12.1%, respectively, which is consistent with the findings of previous research (Buchanan et al., 2008; Bicici et al., 2012). Ruoni (2021) also found that KT can improve ankle proprioception and lower limb dynamic balance ability in CAI patients in a short period. However, past findings were generally inconsistent. Yazici et al. (2015) found that KT intervention in the ankle joint of stroke patients could not change their static balance. Esposito et al. (2021) found that KT could not improve the static and dynamic balance of healthy semiprofessional football players. The varying results may be caused by different experimental subjects or intervention methods. In this study, the KT intervention was able to improve balance ability, which may be explained by the following: after KT is applied to the skin surface, sensory input information of the skin is generated, and proprioceptors may appear as collateral injury after ankle injury. Furthermore, the use of KT can stimulate skin effectors to enhance sensory afferent information; consequently, additional sensory and perceptual pathways are formed in the central nervous system, and the path of the reflex arc is increased, which improves the central nervous control of the periphery whilst enhancing stability (Cheng et al., 2017; Wang et al., 2018). Jackson et al. (2016) also proposed the application of KT to increase proprioceptive function and improve balance performance. The results of this research indicate that balance scores can be significantly improved compared with before intervention, especially when visual and vestibular perception is unfavourable, confirming the conclusions of Ruoni (2021) and Jackson et al. (2016).

### 4.3 Limitations

This study, which entailed a small sample with a no-placebo control, encountered limitations. Future researchers may use more subjects to compare differences in age, sex and other factors. In addition, different taping methods have not yet been applied to patients to determine whether different taping methods have varying effects on college basketball players with FAI. Future studies may also consider adding other measures (i.e., neuromyography) and implementing longer follow-up investigations. Non-etheless, the mechanism related to KT’s improvement of muscle strength and the balance ability of FAI patients must be explored to generate more indicators.

## 5 Conclusion

KT can increase the isokinetic strength of the ankle dorsalis and plantaris flexion in college basketball players with FAI. The KT intervention presented a negligible effect on lower limb stability in patients with FAI during static balance tests, but it significantly improved the balance ability of FAI patients in the dynamic balance test.

## Data Availability

The original contributions presented in the study are included in the article/supplementary material, further inquiries can be directed to the corresponding authors.
